# Impact of dementia-landscaped therapy garden on psychological well-being– A pilot study

**DOI:** 10.1007/s00702-025-02917-z

**Published:** 2025-04-09

**Authors:** Sandra Jevtic, Max Wittlinger, Sonia Teimann, Jens Wiltfang, Norbert Scherbaum, Jens Benninghoff

**Affiliations:** 1 Center for Geriatric Medicine and Developmental Disorders (ZfAE), kbo-Isar-Amper-Klinikum München Ost, München Ost, Germany; 2 Clinic for Psychiatry and Psychotherapy, LVR-Klinikum Essen, Clinic and Institute of the University, Duisburg-Essen, Germany; 3 Institute for Urban Planning and Urban Development, Advanced Research in Urban Systems (ARUS), University of Duisburg-Essen, Duisburg-Essen, Germany; 4 Department of Psychiatry and Psychotherapy, University of Göttingen, Göttingen, Germany

**Keywords:** Dementia, Therapy garden, Psychological-wellbeing, Severity of dementia, Behavioral and psychological symptoms of dementia (BPSD)

## Abstract

Non-pharmacological interventions are increasingly recognized as first-line therapies for managing dementia symptoms alongside pharmacologic strategies. Among these, therapy gardens and horticultural interventions have emerged as promising adjunctive approaches. This pilot study aimed to evaluate the effects of a six-month dementia-friendly therapy garden intervention on psychological well-being, specifically depression levels, and to determine whether baseline dementia severity predicts treatment success. The study was conducted in a real-world setting, with a final sample of 28 dementia patients. Unlike previous studies, this intervention incorporated multimodal stimulation, including sensory, motor, and cognitive elements. Results indicated a significant reduction in depression, as measured by the Montgomery-Åsberg Depression Rating Scale (MADRS) after six months of intervention (*p* <.05). However, depression scores assessed using the Hamilton Depression Rating Scale (HAM-D) showed only a trend toward improvement but did not reach statistical significance. No improvements were observed at the three-month mark, suggesting that sustained engagement is necessary for measurable benefits. Cognitive function, as assessed by dementia severity, did not show significant improvement, and dementia severity at baseline was not a significant predictor of treatment response. These findings underscore the potential of dementia-friendly therapy gardens to provide meaningful psychological benefits by significantly reducing depression over time. Notably, even individuals with more advanced dementia benefited, challenging the prevailing notion that non-pharmacological interventions are primarily effective in early disease stages. These results highlight the need for further research on the long-term effects and mechanisms underlying garden-based interventions in dementia care.

## Introduction

Dementia today is considered an umbrella syndrome caused by a disease of the brain characterized by impaired higher cognitive functions along with difficulties in motivation, social behaviour and emotional control ranking among the most important global health burdens (e.g. Liu et al. 2024; Wehrmann et al. 2021). According to the World Health Organization dementia is not only the leading cause of dependency and disability among older people, but it is associated as the seventh most important cause of death worldwide: There are 55 million patients currently suffering from dementia tending to increase by around 10 million per year (World Health Organization 2023). This increasing demand for care (Wehrmann et al. 2021) and the associated rise in costs (Bosmans et al. 2023) underscore the urgent need to develop, evaluate, and implement effective and cost-efficient therapeutic approaches. Given the fact that dementia is a progressive, incurable disease leading to death (Smith and Ismail 2021), these therapy methods should ideally slow-down expected worsening of dementia symptoms and thus extent the patients´ lifespan towards longer healthspan, at least in terms of better life quality or emotional well-being.

Pharmacological and non-pharmacological approaches are currently used to treat dementia symptoms (e.g. Hussin et al. 2023; Magierski et al. 2020). As part of pharmacological treatment, various substances are used against characteristic neuropsychiatric symptoms of dementia, such as apathy, agitation, depression, psychotic symptoms and sleep disorders (Magierski et al. 2020). These include antidepressants, antihistamines, typical and atypical antipsychotics, Z-drugs as well as anxiolytics, anticonvulsants and in the case of Alzheimer´s acethylcholine-esterase inhibitors (AChE) donepezil, rivastigmine or galantamine or memantine as NMDA-receptor modulator (Magierski et al. 2020). However, there is only a small number of randomized controlled trials that prove the effectiveness of pharmacotherapy for dementia patients (Magierski et al. 2020). At this point, it is too early to predict the effect of the newly released disease-modifying monoclonal antibodies lecanemab or donanemab (Jessen et al. 2024). In addition, there are serious side effects of the current pharmacotherapy in the context of dementia treatment in general, including the increased risk of mortality in the long-term use (Abraha et al. 2017; Brent 2024; Magierski et al. 2020). Looking at empirical evidence, it has been shown that non-pharmacological treatment methods are more or at least equally effective than pharmacotherapy in the treatment of dementia symptoms (Watt et al. 2021).

As a result, non-pharmacological treatment options are repeatedly recommended as first-line therapy in the treatment of dementia (e.g. Brent 2024; Dyer et al. 2018; Tampi and Jeste 2022). Looking at these interventions in particular, a plethora of different approaches surface. These range from sensory stimulation interventions including therapeutic gardens or music therapy to behavioural management techniques, emotion and cognitive-oriented interventions such as validation and reminiscence therapy as well as interventions including pet and exercise therapy (Abraha et al. 2017). Among these approaches, therapeutic gardens have recently been investigated in a meta-analysis (Wang et al. 2024). Therapy gardens in general are a multimodal treatment approach, which usually includes plant-based activities as well as stimulation of all of the five senses and physical exercises (Abraha et al. 2017; Bourdon and Belmin 2021). This becomes an effective, highly adaptable and empirically proven non-pharmacological treatment option (Wichrowski and Moscovici 2024). Effectiveness of therapeutic gardens for patients with dementia has already been shown: Clinical improvements were found in agitation, cognition, stress, depression, self-consciousness (Murroni et al. 2021) as well as quality of life (Murroni et al. 2021; van der Velde-van Buuringen et al. 2023) and social interactions together with augmented activity (Murroni et al. 2021; Scott et al. 2022). Moreover, a decrease in falls and medication needed, i.e. polypharmacy, could be demonstrated (Murroni et al. 2021). Moreover, in a recent network meta-analysis by Liu et al. (2023), therapeutic gardens were found to be more effective than other non-pharmacological treatments in improving dementia symptoms. Theoretically, these positive effects of therapeutic gardens can be explained by various bio-psycho-evolutionary theories (Murroni et al. 2021).

Overall, therapeutic gardens appear to be a promising non-pharmacological, multimodal intervention for dementia. However, many previous studies have not fully embraced multimodality, instead focusing on singular aspects of garden therapy. Some interventions have been limited to sensory stimulation through plants alone (Collins et al. 2020), passive garden viewing (Goto et al. 2018), or simple interactions such as touching plants and walking (Pedrinolla et al. 2019). This contrasts with current recommendations emphasizing that therapy garden interventions should incorporate a combination of multisensory, motor, cognitive, and horticultural activities (Bourdon and Belmin 2021; Elbasyoni and Gammaz 2023). Studies suggest that multimodal therapy gardens are more effective in addressing behavioral and psychological symptoms of dementia (BPSD) compared to interventions that focus on a single element (Bourdon and Belmin 2021).

The aim of this pilot study was to investigate the impact of a therapy garden developed by landscape architects particularly for dementia patients in their natural environment (Teimann 2015). Unlike earlier approaches this dementia garden was equipped with a variety of elements, which facilitate multimodal non-pharmacological treatment. We examined the effectiveness of the dementia-friendly therapy garden focusing on the psychological well-being of the participating dementia patients. In order to find out whether patients with varying degrees of impairment benefit differently from such a therapy garden, we further examined the severity of dementia as predictor of therapy success. It is important to highlight that, in addition to its psychological focus, this pilot study also placed significant emphasis on landscape architectural elements.

## Methods

Initiated by the urban research program (ARUS) of University of Duisburg-Essen (UDE), our interdisciplinary pilot study was carried out with the Institute for Urban Planning and Urban Development (UDE), the Clinic for Psychiatry and Psychotherapy, LVR-Klinikum Essen, the Theodor Fliedner Foundation, Mülheim and the Center for People in Need of Care, Alfter, Germany.

### Participants

Study subjects were recruited from the village *Das Dorf– Wohnen im Alter* run by the Theodor Fliedner Foundation in Mülheim an der Ruhr, Germany. This village is an inclusive community for people with and without disabilities as well as old people and those in need of care. At the time the participants were recruited, a total of 130 residents with diagnosed dementia lived there. All residents who, in addition to a diagnosed dementia, did not have aphasia and had a sufficient level of locomotor function and resilience to be able to take part in the intervention were included given informed consent from their peers. Patients with clinically significant cognitive and motor deficits were excluded. The final sample of the present study consisted of a total of 28 dementia patients. The age ranged from 61 to 95 years (*mean age* = 81.29, *SD* = 9.32). The majority of the test subjects was female (*n* = 22; 78.6%). Ethical approval was issued by the Ethics commission of medical faculty of the University of Duisburg-Essen.

## Procedure and design

**Fig. 1:**
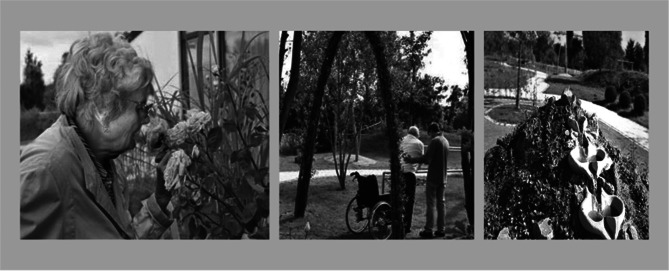
Impressions from the dementia-landscaped garden; Copyright by S. Teimann

The therapy garden was implemented in the village *Das Dorf– Wohnen im Alter* and here, experimental data were collected in the test subjects` natural environment. Based on analyzes of other therapeutic gardens, this landscaped garden was specifically designed to meet the needs and abilities of people suffering from dementia. It consisted of multisensory, interdisciplinary dementia-friendly elements. These included circular paths reflecting the village character, various modules made of plant material for occupational and physiotherapeutic exercises to promote walking safety, muscle strength, motor and coordination skills, as well as raised beds rendering active gardening possible even to villagers with physical limitations. Pavilions were set-up protecting garden visitors from the sun. Orientation in the garden was ensured by several mikado-like robin posts. In order to ensure not only a dementia-friendly design but also a dementia-friendly use of the garden, a total of 70 relatives, volunteers and employees, including social workers and nursing staff, took part in a training program before the start of the intervention. Here, in addition to theoretical knowledge on dementia, they also received information on proper use of the various garden elements (Fig. 1).

**Fig. 2:**
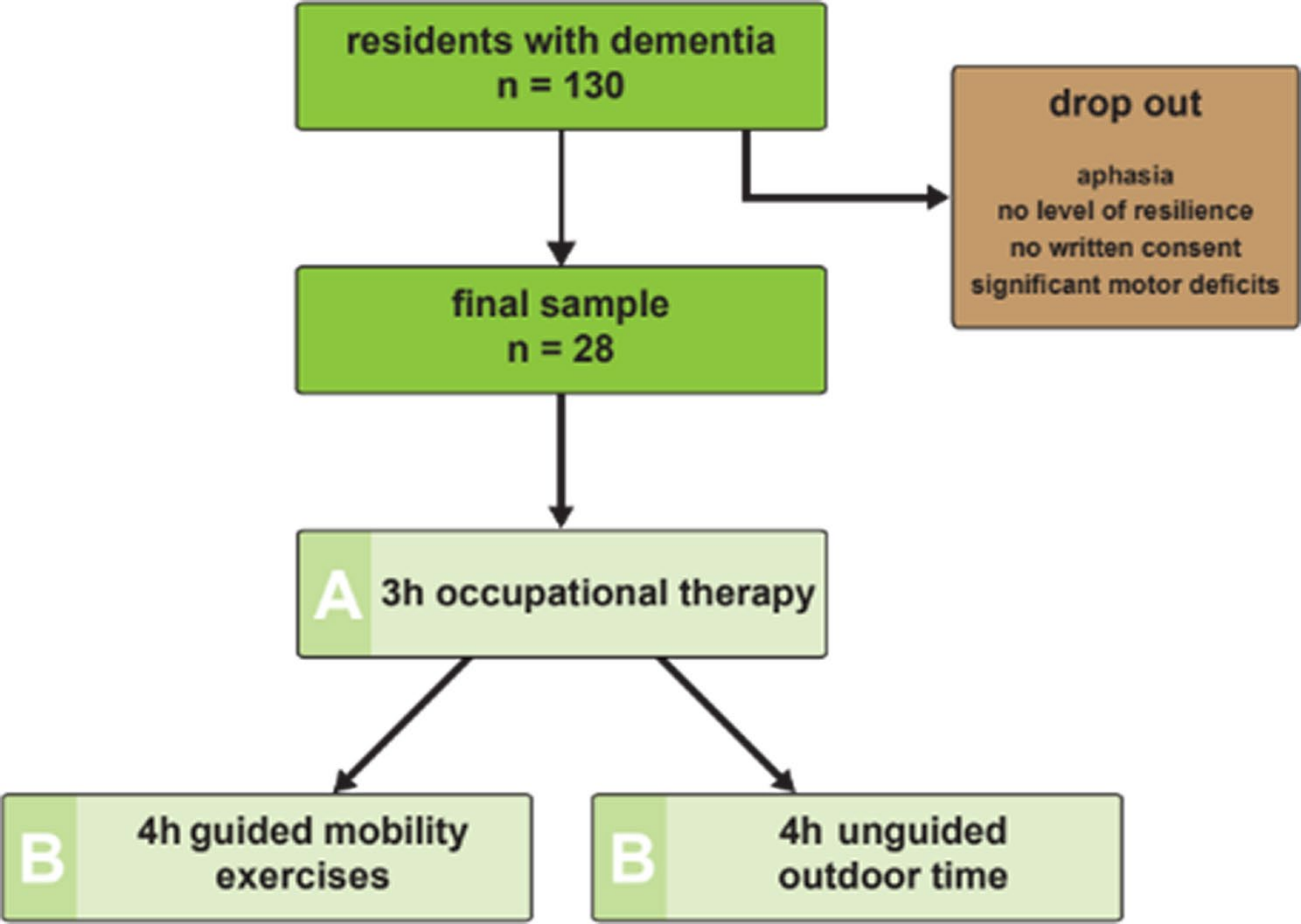
Flowchart describing the process from recruiting study subjects from the initial cohort and why there have been so many potential participants excluded from the study. The final study cohort performed a two-step program: General occupation therapy for 3 h for all subjects. Then, according to the cognitive state additional 4 h under guidance with a special protocol or unguided because of inability to follow suit

Therapy garden intervention took place for a total of six months between March and September. Test subjects received three hours per week of nature-oriented occupational therapy and, depending on the dementia severity, four hours per week of mobility exercises, or they just spent passively four hours in the garden. On average, study subjects took part in the intervention for one hour daily, accompanied most of the times. To examine changes in psychological well-being and severity of dementia over the course of intervention, data was collected at three measurement time points, before the intervention started (T1), after three months (T2) and after six months of participation, i.e. (T3). However, daily participation in the intervention was voluntary and not always possible for all villagers. Therefore, complete data from the entire sample could not be collected at every measurement point (Fig. 2).

## Neuropsychological tests

Dementia severity was screened by Mini-Mental State Examination– MMSE (Folstein et al. 1975). When measuring changes in psychological well-being, we focused on the participants` depression, which was assessed with the *Montgomery-Asberg Depression Rating Scale* (MADRS; Montgomery and Åsberg 1979) as well as the *Hamilton Depression Scale* (HAM-D; Hamilton 1960). The MADRS is an external assessment questionnaire with ten items that are answered on a seven-point Likert scale ranging from 0 to 6. By summing up the ten item values, an overall score is obtained, with higher values indicating a higher degree of depression. Regarding the psychometric properties a good internal consistency of the MADRS is reported in literature with *ɑ* = 0.85 (Levin et al. 2015). Since it is recommended to use multiple measurements to determine depression (Seemüller et al. 2023), we also used the HAM-D. The HAM-D is an external assessment scale, which also showed similar internal consistency (Seemüller et al. 2023). In the version we used, it consisted of a total of 20 items that were answered on Likert scales ranging from 0 to 2, from 0 to 3 or from 0 to 4. Higher total values indicated a higher degree of depression. Since the MADRS is considered to be a suitable questionnaire for assessing treatment effects in homogenous samples such as ours (Seemüller et al. 2023), we used the MADRS values of the test subjects after the six-month intervention as an indicator of treatment success.

## Statistical analysis

Statistical analysis was carried out with the statistical program R, version 4.1.1 (R Core Team 2021). To test the effectiveness of the dementia garden, we used dependent samples t-test. We also calculated t-tests for dependent samples to examine changes after three months of intervention. To investigate whether the severity of dementia predicts the success of treatment, we carried out a linear regression with severity of dementia at T1 as predictor variable and the MADRS values at T3 as dependent variable.

## Results

**Fig. 3:**
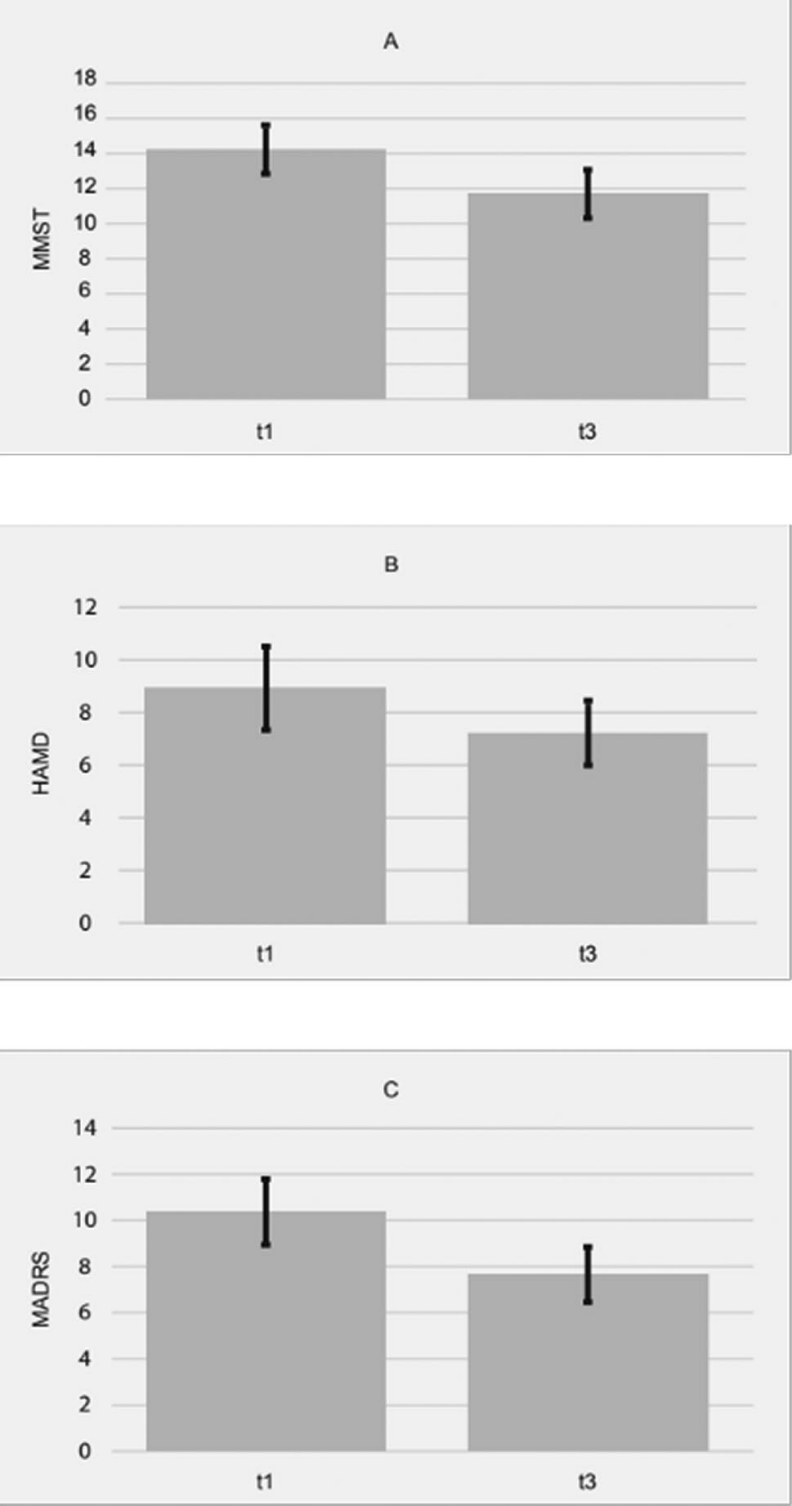
**A**-**C** Values measured at start (t1) and after six months (t3). Ticks indicate SEM. **A**: There was no statistical significance between the MMST values after six months in this cohort (*t*(21) = 3.59, *p* >.05). **B**: No statistiscal differences after six months of the intervention detected by HAM-D, though there was a tendency towards improved HAM-D rating (*t*(12) = 1.75, *p* =.052). **C**: There was a significant difference in terms of amelioration of the MADRS scores (*t*(24) = 1.73, *p* <.05, *d* = 0.35)

Our final sample (*n* = 28) consisted of six patients with mild dementia (21.4%), eleven with moderate dementia (39.3%) and seven with serve dementia (25.0%) before the start of the therapy garden intervention. The MMSE values before the intervention were missing for four participants (14.3%). The average MMSE (Fig. 3A) values increased over the course of the garden intervention given the progressive nature of dementia. The average depression values of the test subjects, on the other hand, improved. In line with these results, we were unable to find any significant improvements after six months of intervention in the MMSE values, (*t*(21) = 3.59, *p* >.05). Even after the first three and last three months of using the dementia garden, the MMSE values did not improve significantly. By contrast, depression measured by MADRS (Fig. 3C) improved statistically significantly after six months of intervention (*t*(24) = 1.73, *p* <.05, *d* = 0.35). In line with this, a marginally significant decrease in depression measured with the HAM-D (Fig. 3B) was found for this time period (*t*(12) = 1.75, *p* =.052). However, again, no significant improvements were found for either the MADRS or the HAM-D values after the first and last three months of intervention. With regard to the prediction of the therapy success, the severity of dementia was not a significant predictor (*F*(1,21) = 0.14, *p* >.05).

## Discussion

The aim of this early pilot study was to evaluate the effectiveness of a dementia-friendly therapy garden incorporating various multimodal landscape elements. Additionally, we examined whether dementia severity at baseline predicted treatment outcomes. Our findings revealed a significant improvement in psychological well-being after six months of intervention, as assessed by the MADRS scale, while no significant cognitive improvements were observed, consistent with the progressive nature of dementia. Notably, no significant changes were detected after three months, suggesting that sustained engagement is necessary for measurable effects. Furthermore, dementia severity did not significantly predict treatment success, indicating that individuals benefitted from the intervention regardless of the severity of their condition.

In line with the results of a previous study by Hewitt et al. (2013), we were unable to demonstrate any significant improvement in cognition as measured by the MMSE after six-months of intervention. By contrast, Pedrinolla et al. (2019) found a significant improvement in the MMSE values after a six-month therapy garden intervention. This discrepancy may stem from methodological differences, since their study included a control group and potentially a different intervention design. Our results suggest that dementia-friendly therapy gardens focusing on emotional well-being may have different therapeutic effects than interventions targeting cognitive function.

In their study Borella et al. (2023) compared the effects of a horticultural activity program combined with cognitive stimulation for dementia patients with those without cognitive stimulation in such a program. The authors were unable to find any significant differences between the two groups in any of the outcome variables, including cognitive symptoms. However, comparable to Pedrinolla et al. (2019), they found differences in cognitive functioning between the therapy garden intervention group and control. These results make clear that the use of therapy gardens in the treatment of dementia can have a positive effect on cognitive functioning, even without improving cognitive abilities. Still, at the same time the results of this study once again underline the importance of comparing therapy effects with a control group. This immediate urge will not be neglected in our upcoming studies on that issue.

Beyond methodological differences, the observed inconsistency in cognitive outcomes may also stem from variations in intervention design. Our findings suggest that dementia-friendly therapy gardens may exert differential effects depending on the specific intervention approach, with our study primarily targeting emotional well-being rather than cognitive function. This underscores the necessity of standardizing interventions, not only to facilitate direct comparisons between different approaches but also to establish clearer associations between intervention characteristics and their respective outcomes.

Regarding cognition, it is important to acknowledge that, given the inherently progressive and incurable nature of dementia, cognitive decline remains an expected trajectory. Thus, a deterioration in cognitive function over time is likely, regardless of participation in a therapy garden intervention, particularly in long-term assessments. However, a slowing-down effect could be achieved. Due to the lack of a control group in our study, this aspect was not further examined. The improvement in psychological well-being after the therapy garden intervention found in the present study is therefore in line with the findings of current reviews (Murroni et al. 2021; Scott et al. 2022; Wang et al. 2024).

Several theoretical frameworks help explain the potential benefits of therapy gardens for dementia patients. The biophilia hypothesis (Wilson 1984) suggests that humans have an innate connection to nature, which may promote psychological well-being.The Stress Reduction Theory (Ulrich et al. 1991) posits that exposure to natural environments can reduce physiological stress markers, such as cortisol levels, thereby improving mood and cognitive function.

Attention Restoration Theory (Kaplan 1995) highlights the cognitive benefits of natural settings, arguing that green spaces provide a “soft fascination” that allows for cognitive replenishment. Recent neurobiological evidence suggests that nature exposure may enhance neuroplasticity and modulate autonomic nervous system activity, potentially slowing cognitive decline (van Praag et al. 2000; Li et al. 2010; de Keijzer et al. 2018). Future studies should explore these mechanisms by incorporating biomarkers (e.g., heart rate variability, salivary cortisol) and neuroimaging techniques to assess structural and functional brain changes.

While various theories attempt to explain the therapeutic effects of garden interventions, a structured prioritization of these frameworks is essential. Instead of treating all theories as equally influential, it is crucial to recognize their complementary rather than competing roles in understanding the benefits of therapy gardens. For example, Stress Reduction Theory and Attention Restoration Theory provide immediate psychological and cognitive explanations, whereas Biophilia Theory offers a broader evolutionary perspective.

Moreover, the neurobiological mechanisms underlying these effects should be integrated with behavioral observations. Theories that emphasize stress modulation (e.g., reduced cortisol, autonomic nervous system balance) are more directly measurable and can be linked to the observed reduction in depression. Thus, future research should prioritize these theories in empirical studies while acknowledging broader conceptual frameworks as supportive rather than primary explanations.

The importance of gardens extends beyond neurobiological and psychological mechanisms. In his landmark book “Gardens: An Essay on the Human Condition”, Robert Pogue Harrison (Harrison, R. P. (2008) explores how gardens serve as spaces of cultural, existential, and philosophical significance. Harrison argues that gardens are not merely restorative spaces but symbolic realms where human civilization negotiates its relationship with nature, memory, and mortality. This aligns with the idea that therapy gardens do not only provide sensory stimulation but also serve as sites of meaning-making, social connectedness, and personal reflection.

Harrison’s work suggests that the therapeutic potential of gardens may arise from their role as sanctuaries that mediate between order and chaos, offering patients a structured yet naturalistic environment that fosters emotional well-being. This perspective provides a deeper understanding of why dementia-friendly therapy gardens may have unique benefits beyond cognitive and affective improvements. Rather than viewing therapy gardens solely as interventions, we may conceptualize them as ‘spaces of care’ that facilitate a continuity of self, even in the face of progressive cognitive decline.

Integrating Harrison’s insights, future research should explore how the symbolic and existential aspects of therapy gardens influence patient outcomes. This could be achieved through qualitative research approaches that examine patient narratives, caregiver perspectives, and the lived experience of garden spaces as therapeutic environments.

Improvement in psychological well-being not only has positive effects on those affected. Additionally, it was shown that the positive effect of a therapy garden on the dementia patients was associated with a stress reduction in the family and staff (Edwards et al. 2012). We assume that relatives, peers, and nursing staff also benefit from improved psychological well-being. Moreover, there is an expected reduction in administration of psychotropic drugs, such as antidepressants. Future studies of our research will also focus on that issue, i.e. reduction of polypharmacy.

It is noteworthy that we found an improvement in psychological well-being after six months, but not after three months underlining a current meta-analysis by Wang et al. (2024), who also showed that the mere duration of therapy garden interventions has a significant influence on their effectiveness. The authors showed that interventions lasting longer than six months were less effective than shorter ones. Still, it might be interesting to see what would happen after more than six months of intervention. Taken together, these findings illustrate the importance of multiple measurement points when examining the effectiveness of implementing garden therapy for patients suffering from dementia.

The distinction between MADRS and HAM-D results warrants further discussion. The MADRS primarily assesses core depressive symptoms, whereas the HAM-D includes somatic and vegetative symptoms, which may explain the observed differences. In connection with this, it could be shown that the results of the two questionnaires differ, particularly in trials with a small sample size like this study. Consequently, differences between the two measures could be minimized with a larger sample size (Guizzaro et al. 2020).

This study has several limitations that should be acknowledged. First, the small sample size (*n* = 28) restricts the statistical power of our findings. Given the heterogeneity of dementia etiology, future studies should aim for larger sample sizes to ensure subgroup analyses (e.g., Alzheimer’s disease vs. vascular dementia). Second, the absence of a control group prevents us from drawing strong causal inferences regarding the efficacy of the therapy garden. It remains possible that improvements in well-being were influenced by factors such as increased social interaction, seasonal effects (e.g., exposure to sunlight and fresh air), or caregiver engagement rather than the garden itself. Third, participation bias is a concern, as only residents who met specific inclusion criteria (e.g., absence of severe motor deficits, aphasia) were enrolled. Excluding patients with severe mnestic deficits may have influenced the generalizability of the findings, as dementia is primarily defined by cognitive decline. Future research should consider stratifying participants based on dementia severity to assess differential effects.

Compliance was also a challenge, as participation in the intervention was voluntary. Some participants may have engaged more frequently, which could have contributed to variability in treatment response. Moreover, the lack of long-term follow-up limits our understanding of whether the observed improvements in psychological well-being persist over time or diminish once the intervention ends. Finally, we did not control for potential confounding factors such as medication changes, nutritional status, or comorbid psychiatric conditions, which may have influenced depressive symptoms.

A key concern in such studies is suitability of the MMSE for detecting short-term cognitive changes. The MMSE is a widely used screening tool but may lack sensitivity to subtle cognitive improvements, particularly over brief intervention periods. Alternative assessments, such as the Montreal Cognitive Assessment (MoCA) or domain-specific neuropsychological tests, may provide more precise measurements of cognitive change.

In addition, while we focused on depression as an indicator of emotional well-being, we recognize that depression and emotional well-being are distinct constructs. Depression scales (MADRS, HAM-D) primarily assess negative affect, whereas broader well-being measures (e.g., WHO-5 Well-Being Index) capture positive emotional states. Future studies should incorporate both types of measures to provide a more comprehensive assessment.

Moreover, behavioral symptoms such as agitation, aggression, and sleep disturbances were not directly measured, even though these are common targets of non-pharmacological dementia interventions. Incorporating behavioral symptom assessments (e.g., Neuropsychiatric Inventory) would allow for a more nuanced evaluation of therapy garden effects beyond mood improvements.

## Conclusion

This study highlights the potential of dementia-friendly therapy gardens as a non-pharmacological intervention to improve psychological well-being. Despite methodological limitations, our findings suggest that therapy gardens can provide meaningful benefits regardless of dementia severity. By refining study designs and integrating neurobiological assessments, future research can further elucidate the mechanisms underlying these therapeutic effects.
